# The Coronavirus Disease 2019 Pandemic in Taiwan: An Online Survey on Worry and Anxiety and Associated Factors

**DOI:** 10.3390/ijerph17217974

**Published:** 2020-10-30

**Authors:** Wei-Hsin Lu, Nai-Ying Ko, Yu-Ping Chang, Cheng-Fang Yen, Peng-Wei Wang

**Affiliations:** 1 Department of Psychiatry, Ditmanson Medical Foundation Chia-Yi Christian Hospital, Chia-Yi City 60002, Taiwan; wiiseen@gmail.com; 2 Department of Nursing, College of Medicine, National Cheng Kung University, Tainan 70101, Taiwan; nyko@mail.ncku.edu.tw; 3 School of Nursing, The State University of New York, University at Buffalo, New York, NY 14214-3079, USA; yc73@buffalo.edu; 4 Department of Psychiatry, School of Medicine, College of Medicine, Kaohsiung Medical University, Kaohsiung 80708, Taiwan; p03132006@gmail.com; 5 Department of Psychiatry, Kaohsiung Medical University Hospital, Kaohsiung 80708, Taiwan

**Keywords:** COVID-19, worry, anxiety

## Abstract

This study explored the associations of individual factors (demographic characteristics, self-confidence in responding to the coronavirus disease 2019 (COVID-19), and self-rated physical and mental health) and environmental factors (perceived confidence in COVID-19 management by the regional government and adequacy of resources and support available to address the COVID-19 pandemic) with worry toward COVID-19 and general anxiety among people in Taiwan. The Chi-square was used to compare difference for worry and anxiety among categorical variables. The logistic regression was used to examine the associations between worry as well as anxiety and individual as well as environmental factors. In total, 1970 respondents were recruited and completed an online survey on worry regarding COVID-19, general anxiety during the pandemic, and individual and environmental factors. In total, 51.7% and 43.4% of respondents reported high levels of worry toward COVID-19 and general anxiety, respectively. Exhibited worse self-rated mental health, lower self-confidence in COVID-19 management, and insufficient mental health resources were significantly associated with high levels of both worry toward COVID-19 and general anxiety. Lower perceived confidence in COVID-19 management by the regional government was associated with a higher level of worry toward COVID-19. Lower perceived social support was associated with a higher level of general anxiety during the COVID-19 pandemic. The results showed that high levels of worry toward COVID-19 and general anxiety were prevalent during the outbreak. This suggests health care providers need additional surveillance of worry and anxiety during the pandemic. Multiple individual and environmental factors related to worry toward COVID-19 and general anxiety were identified. Factors found in the present study can be used for the development of intervention programs, supportive services, and government policy to reduce worry and anxiety during the COVID-19 pandemic

## 1. Introduction

### 1.1. The Psychological Response during Pandemic

At the end of December 2019, people developed life-threatening pneumonia in Wuhan, China. A novel coronavirus, coronavirus disease 2019 (COVID-19), was identified as the source of the infection. The number of reported cases has subsequently increased rapidly across the world. On 30 January 2020, the World Health Organization (WHO) declared the disease a public health emergency of international concern [1].

The neuropsychiatric link between the outbreak of acute respiratory infections and mental disorders can be traced back to the influenza and severe acute respiratory syndrome (SARS) pandemics that occurred years ago [2]. One core neuropsychiatric response during a pandemic is anxiety [3]. During the COVID-19 pandemic, anxiety can develop in healthy individuals as well as in those with preexisting mental health conditions [4]. Social distancing and quarantine are essential methods to prevent the spread of COVID-19 [5,6]; however, both may result in anxiety because the confined people are separated from their loved ones, and their personal liberty is restricted [7,8]. A study of the influenza pandemic among health workers showed that anxiety may preclude health behaviors [9]. Anxiety may also be a risk factor for the worsening of mental illnesses [10] and suicidal behaviors [11]. Moreover, negative societal behaviors, such as societal rejection and discrimination, are often driven by anxiety [4]. Therefore, anxiety during the COVID-19 pandemic is a crucial mental health issue. Wang el al. reported people in China experience anxiety during early outbreak of COVID-19. Examining the anxiety after the early outbreak of COVID-19 is important for us to understand how level of anxiety is when COVID-19 was declared pandemic by WHO [1].

Disease-related worry is the another common affective reaction to an illness [12]. A study investigating the sociocultural features of the influenza pandemic showed that worry toward influenza was the most commonly reported symptom across various sociocultural populations [13]. Disease-related worry has a crucial role in disease management and prevention, particularly for contagious disease management. Disease-related worry is a psychological response to disease and positively affects disease management and prevention by motivating individuals to participate in health promotion activities [6,7,8]. However, disease-related worry can also exert a detrimental effect. For example, it may result in stigmatization, which can have a negative effect on disease management and is associated with the development and worsening of mental disorders [14,15]. An online survey in which most respondents were from the Western world showed people experienced worry in March. Few studies explored the worry toward COVID-19 in the Eastern world which was an area more severely threated by COVID-19 at that time [16]. Therefore, worry toward COVID-19 in populations who were severely threated by the pandemic merits examination.

### 1.2. Factors Related to Anxiety and Worry during the Pandemic

Many factors, including demographic, psychosocial, and environmental factors, may influence people’s worry and anxiety. Tahmassian et al. reported self-confidence is a factor that can predict the level of worry and anxiety [14]. Shah et al. argued that psychosocial support and resources are important to psychological response, for example anxiety and worry, during the pandemic [2]. The study in China showed that the level of anxiety may vary with demographic characteristics, self-rated physical and mental health, and perceived confidence in regional government in responding COVID-19 [17]. In addition, trust in government can also be a factor related to disease-related worry during infectious illnesses [18]. Mertens et al. found demographic factors and self-rated health can predict a level of worry in normal people [16]. A study for Middle East respiratory syndrome (MERS) showed demographic factors and self-rated health may be associated with disease-related worry [19]. Identifying factors associated with worry toward COVID-19 and general anxiety may predict people who are at risk of high level of worry and anxiety. Factors that are related to cause worry toward COVID-19 and general anxiety may provide knowledge for the design of prevention and intervention programs in reducing worry and anxiety for current and future respiratory infection disease (RID) outbreaks. However, the worry and anxiety about COVID-19 in the population in Taiwan and factors related to worry and anxiety have not been extensively studied. Several noteworthy actions and responses conducted by the National Health Command Center of Taiwan—including border control, resource allocation, case identification, quarantine of suspected cases, proactive detection, reassurance and education of the public, and formulation of policies in relation to schools and childcare—have so far successfully alleviated the crisis and contained the pandemic in Taiwan [20]. Therefore, it is important to examine worry and anxiety as well as associated factors in a successful disease contain area after the WHO declared the coronavirus outbreak a pandemic.

### 1.3. Aims of the Study

This study explored the associations of individual factors (demographic characteristics, self-confidence in responding to COVID-19, and self-rated physical and mental health) and environmental factors (perceived confidence in COVID-19 management by the regional government, perceived social support and adequacy of resources and support available for the COVID-19 pandemic) with worry toward COVID-19 and general anxiety among people in Taiwan.

## 2. Materials and Methods

### 2.1. Participants

Participants were enrolled through a Facebook advertisement between April 10 and April 20, 2020. The inclusion criteria were age ≥20 years and residence in Taiwan. The Facebook advertisement included a headline, main text, pop-up banner, and link to the research questionnaire website. We designed the advertisement to appear in the Facebook users’ “news feeds,” which are continually updated lists of updates from advertisers and users’ connections (such as friends and the Facebook groups that they have joined). Our advertisement targeted only users’ news feeds as opposed to other Facebook advertising locations (e.g., the right column), because news feed advertisements are the most effective approach for the recruitment of research participants [21]. We targeted advertisements to Facebook users by location (Taiwan) and language (Chinese), where Facebook’s advertising algorithm determined the users to which our advertisements should be displayed. To ensure the recruitment of health care workers, we also posted the link for the Facebook advertisement to LINE (a direct messaging app) and to Facebook groups joined by health care workers. The Facebook users in Taiwan total 19,010,000 which accounts for 79.7% of population [22]. The sample size needed in present study is 1537.

The Institutional Review Board of Kaohsiung Medical University Hospital (KMUHIRB-EXEMPT (I) 20200011) approved this research and waived the need for informed consent because participation was voluntary and survey responses were anonymous. No incentive was provided for participation. Links to information on COVID-19 from the Taiwan Center for Disease Control, Kaohsiung Medical University Hospital, and the Medical College of National Cheng Kung University were prepared for participants to learn more about COVID-19.

### 2.2. Measures

#### 2.2.1. Demographic Variables

Data on respondents’ sex (female and male), age, and education level were collected. Respondents with qualifications of high school level or lower and respondents with qualifications of university level or higher were classified into low- and high-level education groups, respectively. Respondents were also asked whether they were health care workers.

#### 2.2.2. Worry toward COVID-19

We used the following question from the study of Liao et al. [12] to assess how much worry toward COVID-19 was experienced by respondents: “In the past week, have you ever worried about catching COVID-19?” Ratings from 1 (*no*) to 5 (*constantly worried*) were provided for this question. According to Liao et al. [12], participants with ratings of 2.5 or more exhibit a high level of worry toward COVID-19.

#### 2.2.3. General Anxiety

General anxiety in the preceding week was measured using a previously validated state anxiety scale from the State-Trait Anxiety Inventory (STAI), wherein respondents rate their feelings in response to 10 general statements; these statements establish whether respondents feel rested, contented, comfortable, relaxed, pleasant, anxious, nervous, jittery, highly strung, or overexcited and “rattled” [12,23,24]. For each question, a rating of 1 (*not at all*), 2 (*sometimes*), 3 (*moderately so*), or 4 (*very much so*) was provided. Statements reflecting positive feelings were reverse coded. A higher total score for the 10 items represents greater general anxiety. The Cronbach’s α for this section was 0.921. According to Liao et al. [12], participants with ratings of 3 or more exhibit high levels of general anxiety during the COVID-19 pandemic.

#### 2.2.4. Perceived Social Support

Three questions from Tardy’s study were used to assess levels of perceived social support [25]: “How satisfied are you with the support you received from your (1) families, (2) friends, and (3) colleagues during the past week?” Ratings from 0 (*extremely unsatisfied*) to 4 (*extremely satisfied*) were given for each question. The total score for the three questions indicates the level of perceived social support. Higher scores represent better perceived social support.

#### 2.2.5. Self-Confidence in Responding to COVID-19 and Perceived Confidence in COVID-19 Management by the Regional Government

We used the following questions from a questionnaire on the risk perception for infectious disease outbreaks [26] to assess how self-confident respondents were in their ability to *responding to* COVID-19: “How confident are you about coping with COVID-19?” We also used a question to assess respondents’ perceived confidence in COVID-19 management by the regional government: “How confident are you regarding COVID-19 management by the government of the region where you live?” Ratings from 1 (*not confident at all*) to 5 (*very confident*) were provided for these two questions. We used median as the cut-off point [27]. The median scores for both questions were 2. Therefore, people who have score more than 2 were classified as high self-confidence in responding to COVID-19/high perceived confidence in COVID-19 management by the regional government.

#### 2.2.6. Self-Rated Physical and Mental Health

The Self-Perceived Health Questionnaire, developed by Ko et al. [28], contains four questions, with the first two questions assessing participants’ self-rated physical and mental health compared with other people’s health in the preceding week and the last two questions assessing participants’ self-rated physical and mental health compared with other people’s health before the COVID-19 outbreak. Ratings from 0 (*much worse*) to 4 (*much better*) were provided for each question. The sum of the scores for the first and third questions indicates self-rated physical health, and the sum of the scores for the second and fourth questions indicates self-rated mental health. A higher total score indicates better physical and mental health.

#### 2.2.7. Adequacy of Resources and Support for the COVID-19 Pandemic

We estimated participants’ perceptions regarding adequacy of resources and support for the COVID-19 pandemic by using four questions from the WHO Guidance Document [29]: “During the COVID-19 pandemic, did you receive enough of the following resources or support: (1) basic equipment for everyday protection, such as masks and hand sanitizers, (2) financial resources, (3) medical resources, and (4) mental health resources?” The responses to the questions were categorized as *insufficient* and *sufficient*.

### 2.3. Statistical Analysis

Data analysis was performed using SPSS 22.0 statistical software (SPSS Inc., Chicago, IL, USA). The chi-square was used to compare rates of high worry toward COVID-19 and high general anxiety among categorical variables. Pearson’s correlation coefficient was used to examine the correlations of worry toward COVID-19 and general anxiety with continuous variables. The associations of demographic characteristics, self-confidence in coping with COVID-19, perceived confidence in COVID-19 management by the regional government, self-rated physical and mental health, and adequacy of supplies for the COVID-19 pandemic with rates of high worry toward COVID-19 and high general anxiety were examined by using logistic regression analysis. A two-tailed *p* value of <0.05 indicated statistical significance.

## 3. Results

### 3.1. Participant Variables and Level of Worry and Anxiety

In this study, 2031 respondents completed the online questionnaire. After 61 respondents with missing data were excluded, the data of 1970 respondents (665 male and 1305 female respondents) were analyzed. Table 1 shows demographic characteristics, worry toward COVID-19 and general anxiety, self-confidence in coping with COVID-19, perceived confidence in COVID-19 management by the regional government, levels of self-rated physical and mental health, and the adequacy of resources and support available during the COVID-19 pandemic. According to cut-off points which were used by Liao et al. [12], the rates of high levels of worry toward COVID-19 and high levels of general anxiety during the COVID-19 pandemic were 51.7% and 43.4% in our study, respectively.

Table 2 shows the differences in the rates of high worry toward COVID-19 and high general anxiety across demographic characteristics and adequacy of resources and support. The results indicate higher rates of high worry toward COVID-19 and of high general anxiety among female respondents than among male respondents. No significant difference was found in the rates of high worry toward COVID-19 and high general anxiety between respondents with various education levels. Respondents who evaluated the resources and support available for the COVID-19 pandemic to be insufficient exhibited higher rates of high worry toward COVID-19 and high general anxiety during the COVID-19 pandemic than those who evaluated their resources and support to be sufficient. People who had low self-confidence in coping with COVID-19 and perceived confidence in COVID-19 management by the regional government experienced significantly differences in rates of high worry toward COVID-19 and of high general anxiety during the COVID-19 pandemic.

### 3.2. Factors Related to the Level and Rate of Worry and Anxiety

Table 3 shows the correlations of age, self-rated physical and mental health, and social support with worry toward COVID-19 and general anxiety. The results indicate that worse self-rated physical and mental health and lower perceived social support were significantly related to higher levels of worry toward COVID-19 and general anxiety. In addition, younger age was significantly associated with a higher level of general anxiety during the COVID-19 pandemic.

Table 4 shows the results of logistic regression analysis of the odds ratios to worry toward COVID-19 and general anxiety during the COVID-19 pandemic. The results indicate that health care worker status, worse self-rated mental health, lower self-confidence in responding to COVID-19, lower perceived confidence in COVID-19 management by the regional government, and insufficient mental health resources were significantly associated with higher levels of worry toward COVID-19.

Health care workers and participants with worse self-rated mental health, lower perceived social support, lower self-confidence in responding to COVID-19, and insufficient mental health resources showed significantly higher levels of general anxiety during the COVID-19 outbreak.

## 4. Discussion

### 4.1. Levels of Worry and Anxiety during the Outbreak

High level of worry toward COVID-19 was an important mental health issue during the pandemic because more than half experienced this problem. A longitudinal study in Hong Kong during the 2009 influenza pandemic showed that between 40% and 60% of individuals exhibited significant levels of worry toward infection during the outbreak [30]. Ro et al. using the same measurement of worry as the present study found that 36.3%–59.6% of respondents reported a high level of worry toward MERS during the outbreak of the 2015 Middle East respiratory syndrome (MERS) in Korea [19]. The results of the present study and of other have studies indicate that high levels of worry toward RID were prevalent during pandemics across various countries. Studies have reported that high levels of worry toward infection are associated with psychological distress, physical health issues, and stigma [13,31,32]. Therefore, health professionals must provide appropriate psychological interventions for reducing worry [33].

Our study found that the high general anxiety is also prevalent for people during the COVID-19 pandemic. A cross-sectional study showed a high level of STAI-assessed general anxiety during the 2009 influenza A (H1N1) pandemic in Turkey [34]. Research has also found that people reported an elevated level of anxiety during the threat of the SARS pandemic [35] and continued to experience high levels of anxiety 30 months after the SARS outbreak [36]. The previous study in China during the early outbreak of the COVID-19 pandemic reported moderate-to-severe anxiety among 28.8% of participants [17]. Islam et al. also reported anxiety is prevailing in Bangladesh during COVID-19 pandemic [37]. The results of this study and of other studies indicate that the high level of anxiety is prevailing during RID pandemics. A high level of anxiety is related to the decreased adoption of precautionary measures and increased maladaptive behaviors [38,39]. Therefore, health professionals must include anxiety assessment in their routine screening during RID pandemics.

### 4.2. Factors Related to Worry and Anxiety during the Outbreak

In addition to examination of psychological and behavioral responses to RID pandemics [40,41], examination of factors related to worry and anxiety during RID pandemics can provide knowledge for the development of subgroup-specific prevention and intervention strategies. The present study found that multidimensional individual and environmental factors were related to worry toward COVID-19 and general anxiety during the COVID-19 pandemic.

The results indicated that high worry toward COVID-19 and general anxiety were more prevailing among health care workers rather than among non–health care workers. Studies have reported that high levels of worry of being infected among health care workers may be associated with intended absenteeism and decreased social interaction [42,43]. Meanwhile, our result was in line with the previous study in China that showed heath workers may experience more anxiety [44]. Mohindra et al. suggested that increased knowledge of inevitable infection, supportive colleagues, and measures to reduce frustrations with patient care can decrease health workers’ worry during the COVID-19 outbreak [45].

A community study showed that women have higher levels of worry and general anxiety than men across three age cohorts [46]. In the present study, female respondents experienced more general anxiety than male respondents during the COVID-19 pandemic in the univariate analysis. The gender difference was not significant after introducing effects of other factors. This implied that the association between gender and worry as well as anxiety was adjusted by other factors. Meanwhile, further study is required to examine whether gender differences in worry and anxiety levels during the COVID-19 pandemic originate from gender differences in anxiety before the pandemic or from gender differences in psychological reactions to the COVID-19 pandemic.

The present study found that both worse self-rated mental health and lower self-confidence in coping with COVID-19 were associated with higher rates in high general anxiety and worry toward COVID-19. The associations might be bidirectional. Experts indicate that worse mental health may predict worse psychosocial outcomes [47]. Hao et al. found that people with mental illness may experience more worry, anxiety, and suicidal ideation and develop other mental illnesses than people without mental illness during the COVID-19 pandemic [48]. Researchers have also found that poor self-rated health status is significantly associated with greater psychological effects from the outbreak and with higher levels of stress, anxiety, and depression [17]. The review study of Bish et al. concluded that self-confidence is associated with precautionary behavior during RID epidemics or pandemics [49]. The aforementioned poor mental health and low self-confidence may impede people’s motivation and ability to use practical protective measures against COVID-19. A lack of timely and efficient strategies to protect against COVID-19 may further increase people’s worry toward the disease and their general anxiety [17]. High levels of worry and anxiety might also compromise people’s cognitive function, perceptions of mental health, and confidence in their ability to manage daily affairs, including coping with COVID-19. Therefore, interventions for reducing worry and anxiety as well as for improving self-confidence and mental health are critical.

Regarding environmental factors, our results found that perceived confidence in COVID-19 management by the regional government was related to lower rates of the high level of worry. A study of the 2009 H1N1 pandemic in the Netherlands also found that trust in the government was associated with the public’s intention to adopt protective measures [50]. Adopting protective measures may decrease worry toward infectious illness. The present study also found that insufficient mental health resources were associated with more high levels of worry and anxiety. Betty et al. indicated that mental health services are essential for reducing the psychological effects of COVID-19 [47]. This result provided evidence supporting that sufficient mental health resources are crucial during RID epidemics or pandemics [6].

Perceived social support is a significant protective factor for general anxiety during COVID-19. Studies have described that perceived social support is a protective factor for anxiety [51], and that it has a central role in anxiety reduction [52]. However, members of the public were asked to observe social distancing and reduce social interaction. Means by which people interact and provide social support to others may be interrupted during the pandemic. Health professionals and policy makers should develop alternative approaches such as video and telephone visits to provide social support for the public, particularly for those quarantined during the pandemic.

### 4.3. Limitations

The present study has several limitations. First, although Facebook is a promising research method for recruiting participants by targeting the general public during fast-spreading infectious disease outbreaks [53], Facebook users may not be representative of the population. A review of a study that recruited participants through Facebook reported a bias in favor of women, young adults, and people with higher education levels and incomes [54]. Second, the level of general anxiety was self-reported but not evaluated by mental health professionals. Third, the cross-sectional study design limited the possibility of determining a causal relationship of worry and anxiety with self-rated mental health and confidence in COVID-19 management. Fourth, further studies explore other factors which are not included in the present study and may associate with disease-related worry, and anxiety is warranted to help us get a more comprehensive understanding for factors associated with diseased-related worry and anxiety during COVID-19.

## 5. Conclusions

This study found high anxiety and worry toward the COVID-19 among respondents in Taiwan during the pandemic were prevalent. The result highlights the urgency for enhancing health care professionals’ planning for and competence in relation to the detection and management of infectious disease outbreaks’ psychological effects. We found that poor self-rated mental health, low self-confidence in coping COVID-19, and insufficient mental health resources and heath care works were the risk factors for high worry and anxiety. Factors found in the present study can be used for the development of psychological interventions and government policy to reduce worry and anxiety during the COVID-19 pandemic. There are factors which were not examined in present study. Therefore, future lines of research could be carried out to complement this study.

## Figures and Tables

**Table 1:** Descriptive statistics for demographic characteristics, worry toward COVID-19, general anxiety, self-confidence in coping with COVID-19, perceived confidence in COVID-19 management by the regional government, self-rated physical and mental health, and adequacy of resources and support available for the COVID-19 pandemic (*n* = 1970).

Variables	*n* (%)	Mean (SD)
Sex		
Female	1305 (66.2)	
Male	665 (33.8)	
Age (years)		37.81 (10.8)
Education level		
Low (high school or below)	218 (11.1)	
High (university or above)	1752 (88.9)	
Occupation		
Non-health care worker	1324 (67.2)	
Health care worker	646 (32.8)	
Worry about COVID-19		
Low worry	714 (48.3)	
High worry	1256 (51.3)	
General anxiety		
Low anxiety	1115 (56.6)	
High anxiety	855 (43.4)	
Self-confidence in responding to COVID-19		2.41 (0.84)
Perceived confidence of regional government in coping with COVID-19		2.32 (0.95)
Self-rated physical health		4.15 (1.59)
Self-rated mental health		4.61 (1.75)
Perceived social support		8.59 (2.01)
Basic equipment for protection in daily lives for COVID-19		
Sufficient	1437 (72.9)	
Insufficient	533 (27.1)	
Financial resource		
Sufficient	1464 (74.3)	
Insufficient	506 (25.7)	
Medical resources		
Sufficient	1545(78.4)	
Insufficient	425 (21.6)	
Mental health resources		
Sufficient	1559 (79.1)	
Insufficient	411 (20.9)	

**Table 2:** Differences in the rates of high worry toward COVID-19 and high general anxiety according to demographic characteristics and resources and support.

	Worry about COVID-19			General Anxiety		
	Low Worry N (%)	High Worry N (%)	*p*	Low AnxietyN (%)	High AnxietyN (%)	*p*
Sex						
Female	609 (46.7)	696 (53.3)	0.039	718 (55.0)	587 (45.0)	0.047
Male	343 (51.6)	322 (48.4)		397 (59.7)	268 (40.3)	
Education level						
Low	105 (48.1)	113 (51.8)	0.960	115 (52.8)	103 (47.2)	0.224
High	847 (48.3)	905 (51.7)		1000 (57.1)	752 (42.9)	
Occupation						
Non-health care worker	644 (48.6)	680 (51.4)	0.688	742 (56.0)	582 (44.0)	0.475
Health care worker	308 (47.7)	338 (52.3)		373 (57.7)	273 (42.3)	
Personal protective equipment						
Sufficient	728 (50.7)	709 (49.3)	0.001	867 (60.3)	570 (39.7)	<0.001
Insufficient	224 (42.0)	309 (58.0)		248 (46.5)	285 (53.5)	
Financial resource						
Sufficient	737 (50.3)	727 (49.7)	0.002	898 (54.6)	566 (34.4)	<0.001
Insufficient	215 (42.5)	291 (57.5)		217 (42.9)	289 (57.1)	
Medical resources						
Sufficient	797 (51.6)	748 (48.4)	<0.001	938 (60.7)	607 (39.3)	<0.001
Insufficient	155 (36.5)	270 (63.5)		177 (41.6)	248 (58.4)	
Mental health resources						
Sufficient	813 (52.1)	746 (47.9)	<0.001	990 (63.5)	569 (36.5)	<0.001
Insufficient	139 (33.8)	272 (66.2)		125 (30.4)	286 (69.6)	
Self-confidence in responding to COVID-19						
Low self-confidence	375 (36.8)	577 (60.7)	<0.001	476 (46.7)	543 (53.3)	<0.001
High self-confidence	644 (63.2)	374 (39.3)		639 (67.2)	312 (32.8)	
Perceived confidence of regional government in coping with COVID-19						
Low perceived confidence	421 (40.1)	628 (59.9)	<0.001	517 (49.3)	532 (50.7)	<0.001
High perceived confidence	531 (57.57)	390 (42.3)		598 (64.9)	323 (35.1)	

**Table 3:** Correlations of age, self-rated physical and mental health, social support, and confidence in COVID-19 management ability with worry toward COVID-19 and general anxiety.

	Worry about COVID-19		General Anxiety	
	Pearson’s *r*	*p*	Pearson’s *r*	*p*
Age	−0.03	0.241	−0.09	<0.001
Self-rated physical health	−0.19	<0.001	−0.33	<0.001
Self-rated mental health	−0.22	<0.001	−0.49	<0.001
Perceived social support	−0.10	<0.001	−0.30	<0.001

**Table 4:** Odds ratios related to high worry toward COVID-19 and general anxiety during COVID-19 pandemic.

	Worry about COVID-19		General Anxiety	
	Odds Ratios	*p*	Odds Ratios	*p*
Sex ^a^	1.08	0.406	1.07	0.571
Age	1.00	0.504	1.00	0.493
Education level ^b^	0.83	0.229	0.91	0.556
Occupation ^c^	1.24	0.044	1.25	0.044
Self-rated physical health	0.95	0.191	0.98	0.646
Self-rated mental health	0.88	<0.001	0.65	<0.001
Perceived social support	0.99	0.681	0.89	<0.001
Self-confidence in responding to COVID-19 ^d^	0.54	<0.001	0.76	0.018
Perceived confidence of regional government in coping with COVID-19 ^e^	0.70	0.001	0.84	0.136
Personal protective equipment ^f^	1.01	0.914	091	0.460
Financial resource ^d^	1.15	0.277	0.78	0.060
Medical resources ^d^	0.79	0.123	0.99	0.964
Mental health resources ^d^	0.68	0.008	0.47	<0.001

^a^: male as reference; ^b^: high education level as reference; ^c^: non–health care worker as reference; ^d^: low self-confidence as reference; ^e^: low perceived confidence of regional government as reference; ^f^: insufficient as reference.
